# Reverse Engineering Analysis of the High-Temperature Reversible Oligomerization and Amyloidogenicity of PSD95-PDZ3

**DOI:** 10.3390/molecules27092813

**Published:** 2022-04-28

**Authors:** Sawaros Onchaiya, Tomonori Saotome, Kenji Mizutani, Jose C. Martinez, Jeremy R. H. Tame, Shun-ichi Kidokoro, Yutaka Kuroda

**Affiliations:** 1 Department of Biotechnology and Life Science, Tokyo University of Agriculture and Technology, 2-24-16, Naka-cho, Koganei-shi 184-8588, Tokyo, Japan; s227417t@st.go.tuat.ac.jp; 2 Institute of Global Innovation Research, Tokyo University of Agriculture and Technology, 3-8-1, Harumi-cho, Fuchu-shi 183-8538, Tokyo, Japan; t_saotome@vos.nagaokaut.ac.jp; 3 Department of Bioengineering, Nagaoka University of Technology, 1603-1, Kamitomioka-cho, Nagaoka-shi 940-2188, Niigata, Japan; kidokoro@vos.nagaokaut.ac.jp; 4 Graduate School of Medical Life Science, Yokohama City University, 1-7-29 Suehiro, Yokohama 230-0045, Kanagawa, Japan; mizutani@yokohama-cu.ac.jp (K.M.); jtame@yokohama-cu.ac.jp (J.R.H.T.); 5 Department of Physical Chemistry, Institute of Biotechnology, Faculty of Sciences, University of Granada, 18071 Granada, Spain; jcmh@ugr.es

**Keywords:** high-temperature reversible oligomerization, amyloidogenicity, oligomeric interface residues, thermal denaturation, mutational analysis

## Abstract

PSD95-PDZ3, the third PDZ domain of the post-synaptic density-95 protein (MW 11 kDa), undergoes a peculiar three-state thermal denaturation (N ↔ I_n_ ↔ D) and is amyloidogenic. PSD95-PDZ3 in the intermediate state (I) is reversibly oligomerized (RO: Reversible oligomerization). We previously reported a point mutation (F340A) that inhibits both ROs and amyloidogenesis and constructed the PDZ3-F340A variant. Here, we “reverse engineered” PDZ3-F340A for inducing high-temperature RO and amyloidogenesis. We produced three variants (R309L, E310L, and N326L), where we individually mutated hydrophilic residues exposed at the surface of the monomeric PDZ3-F340A but buried in the tetrameric crystal structure to a hydrophobic leucine. Differential scanning calorimetry indicated that two of the designed variants (PDZ3-F340A/R309L and E310L) denatured according to the two-state model. On the other hand, PDZ3-F340A/N326L denatured according to a three-state model and produced high-temperature ROs. The secondary structures of PDZ3-F340A/N326L and PDZ3-wt in the RO state were unfolded according to circular dichroism and differential scanning calorimetry. Furthermore, PDZ3-F340A/N326L was amyloidogenic as assessed by Thioflavin T fluorescence. Altogether, these results demonstrate that a single amino acid mutation can trigger the formation of high-temperature RO and concurrent amyloidogenesis.

## 1. Introduction

The two-state thermal denaturation process is a biophysical hallmark for a natively folded single-domain globular protein. A two-state thermal denaturation process exhibits a sharp endothermic peak as observed by micro-calorimetry [1,2,3,4], and the two-state unfolding can be formally confirmed by thermodynamically analyzing the heat capacity from differential scanning calorimetry (DSC) [5]. Exceptions to the two-state thermal unfolding are observed when a molten globule (MG) state forms upon thermal denaturation [6,7,8,9,10,11], usually under non-physiological conditions (acidic/high salt conditions) [8]. The equilibrium MG is a state where the secondary structure is retained, but the tertiary structure is loosely packed, similar to the kinetic intermediates observed during protein folding [7].

Post-synaptic density-95 protein (PSD-95) is a member of the membrane-associated guanylate kinase (MAGUK) family. Like other MAGUK proteins, PSD-95 consists of three PDZ domains, one SH3 domain, and one guanylate kinase [12,13,14,15]. PDZ3 is the third PDZ domain of PSD-95, containing three α-helices and six β-strands. It is small, globular, and it has a molecular weight of 11 kDa. PDZ3 undergoes a three-state denaturation process. The intermediate state is not a MG state but is an oligomer formed reversibly at temperatures as high as 60 to 70 °C, which we coined high-temperature reversible oligomer (RO) [16,17,18,19].

Recently, we found that the single mutations (F340A and L342A), which do not affect the physicochemical properties of PDZ3 at ambient temperatures, could inhibit the formation of RO and amyloids [16]. Both variants (PDZ3-F340A and PDZ3-L342A) undergoing a two-state unfolding process were designed by replacing hydrophobic residues at the interface of the tetrameric crystal structure of PDZ3 to alanine. Namely, the residues were identified by their large buried surface area (BSA) and accessible surface area (ASA). In particular, F340A mutation inhibited not only high-temperature RO but also amyloidogenesis; however, it would be of interest to understand how a single mutation could induce the formation of an RO state at a high-temperature.

This study applies a reverse engineering strategy to design point mutations to reintroduce high-temperature ROs in PDZ3-F340A. Reverse engineering test our hypothesis on the mechanisms underlying the formation of high-temperature RO in natural sequences. The PDZ3-F340A, which is RO-free, was used as a template protein. We hypothesized RO is produced by a hydrophobic residue on the surface of the monomeric protein and buried at the interface of the tetrameric structure. We thus selected three such hydrophilic residues and replaced them with Leucine (Leu) to enhance the hydrophobic interaction between the monomeric proteins. DSC analysis indicated that two variants unfolded according to the two-state model and did not form high-temperature ROs; however, RO was successfully reintroduced by the N326L mutation, and PDZ3-F340A/N326L was strongly amyloidogenic, confirming a correlation between the appearance of RO and amyloidogenesis.

## 2. Results and Discussion

### 2.1. Reverse Engineering of PDZ3

**Figure 1:**
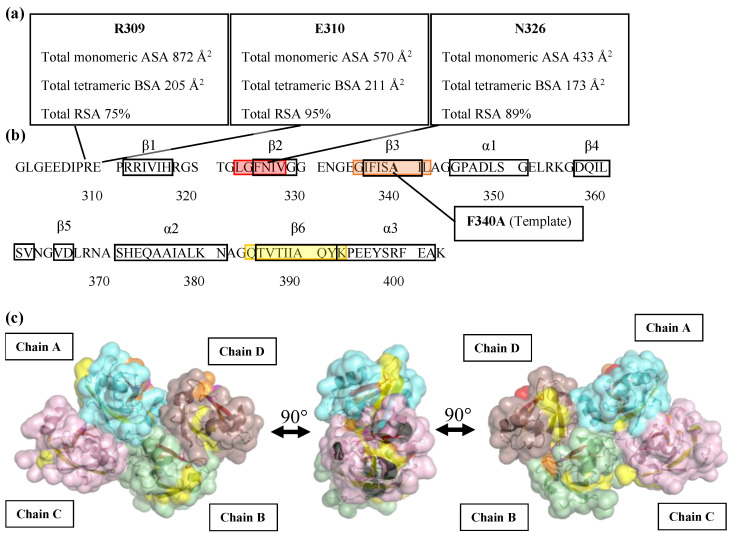
Secondary structure and three-dimensional accessible surface structures of PDZ3-F340A (**a**) Total monomeric accessible surface area (ASA), total tetrameric buried surface area (BSA), and total relative solvent accessible surface area (RSA) values of R309, E310, and N326 residues are shown in square boxes; (**b**) TANGO analysis of β-aggregation (amyloid) prone regions in PDZ3-F340A. The 323–328 residue region crossing the β2 strand is represented in red. The 335–342 residue region crossing the β3 strand is represented in orange. The 384–393 residue region crossing the β6 strand and α-helix 3 at C-terminus is represented in yellow; (**c**) Three images of 90° counter-clockwise views of the PDZ3-F340A tetrameric structure represent the aggregation-prone region on the interface of a tetramer unit cell using Pymol. The color code is the same as in the amino acid sequence.

This study used PDZ3-F340A, which undergoes a two-state thermal denaturation as a template for examining whether one can find point mutations yielding RO at high temperatures. PDZ3-F340A itself was designed by mutating F340A in the wild-type of PSD95-PDZ3, which undergoes a three-state thermal denaturation with the concurrent production of high-temperature RO. The RO-producing mutations are assumed to be hydrophilic residues located on the surface of PDZ3 but at the interface of the tetrameric structural unit. The mutation site was determined by calculating the ASA and RSA using DSSP and by applying the following rules (i) high total monomeric accessible surface area (ASA) and tetrameric buried surface area (BSA), (ii) high total tetrameric relative solvent accessible area (RSA), (iii) non-hydrophobic residue, and, (iv) hydrophobic interaction between a side-chain without a steric clash (Table 1 and Table S1). We thus computed the ASA and BSA of PDZ3-F340A, modeled from the X-ray structure of PSD95-PDZ3-wt (PDB ID: 3I4W) using COOT. A large ASA indicates that the residue is located on the surface of the monomeric protein, while a large BSA indicates that the residue is in the interface of the tetrameric unit cell. We selected three hydrophilic residues based on their RSA and BSA (Figure 1a), namely R309, E310, N326, and visually confirmed their location using PyMol (Figure S1, see Supplementary Materials). Note that the calculation from DSSP was in line with those from PDBePISA [20], which we used in our previous reports [16,21]. Three variants, where the residues mentioned above were individually substituted to a Leucine (Leu) with the aim of inducing high-temperature RO by increasing hydrophobic interactions, were produced in *E. coli*, purified, and characterized as described in the following sections.

### 2.2. Biophysical Characterization of the PDZ3 Variants

**Figure 2:**
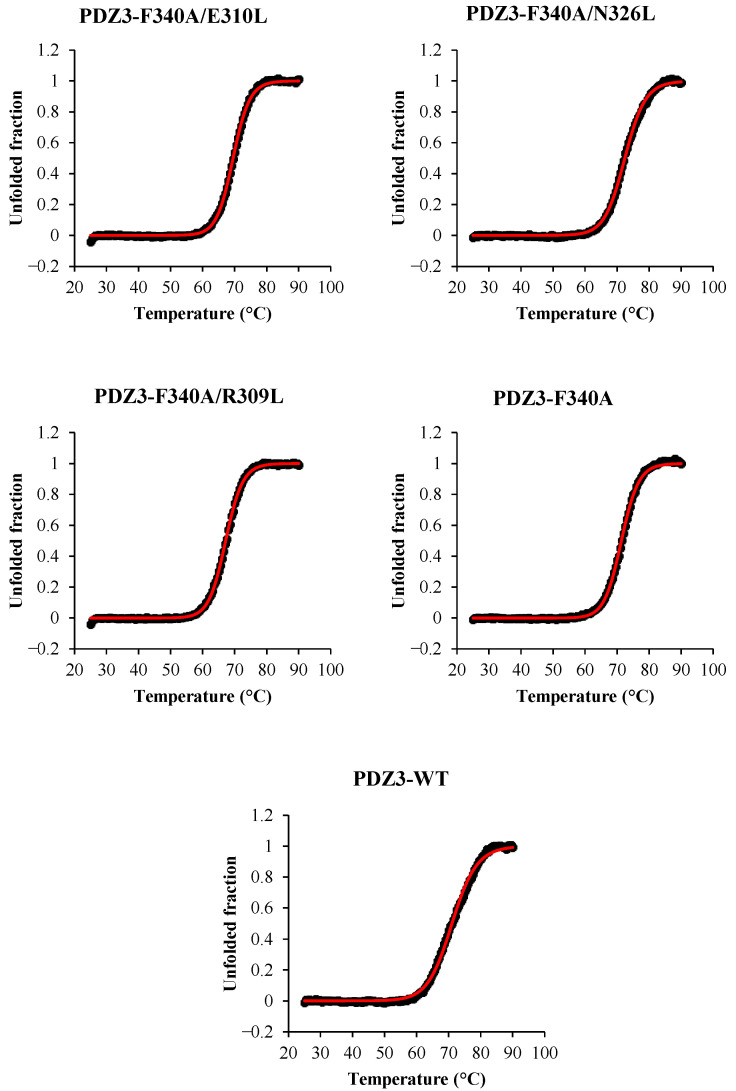
CD thermal denaturation curves of PDZ3 variants at 0.5 mg/mL and pH 7.5 at a scan rate of +1.0 °C/min. The CD values were monitored at 220 nm. Black dots are the experimental data, and red lines are the fitting curves.

The substitution of the candidate residues to Leu did not affect PDZ3 variants’ native structure and physiochemical properties at ambient temperatures. Sedimentation velocity analysis indicated that all PDZ3 variants were monomeric at 25 °C (Table S2, Figure S2). CD spectra of PDZ3 variants showed that the secondary structure contents of PDZ3 variants were identical to that of PDZ3-wt at temperatures up to 60 °C (Figure S3), and the denaturation was reversible as assessed by measuring the spectra after cooling the heated sample to 25 °C. The CD spectra of PDZ3 variants contained similar fractions of antiparallel and parallel β-strands more than α-helix structures in line with the secondary structure content calculated from the X-ray structure of PDZ3-wt (Table S3). The thermal denaturation curves measured by CD at a concentration of 0.5 mg/mL and pH 7.5 were sigmoidal (Figure 2), indicating an apparent two-state denaturation of the secondary structures (N↔D). For discussion, we estimated the apparent thermodynamic parameters, and we calculated the melting temperature (*T*_m_) and van’t Hoff enthalpy (Δ*H*_van’t Hoff_ (*T*_m_)) from the fitting of the CD denaturation curves (Table 2). The apparent Tm of PDZ3-F340A/N326L was the highest at 72.40 °C, whereas R309L and E310L slightly decreased the apparent *T*_m_.

### 2.3. DSC Analysis and Thermodynamic Parameters

**Figure 3:**
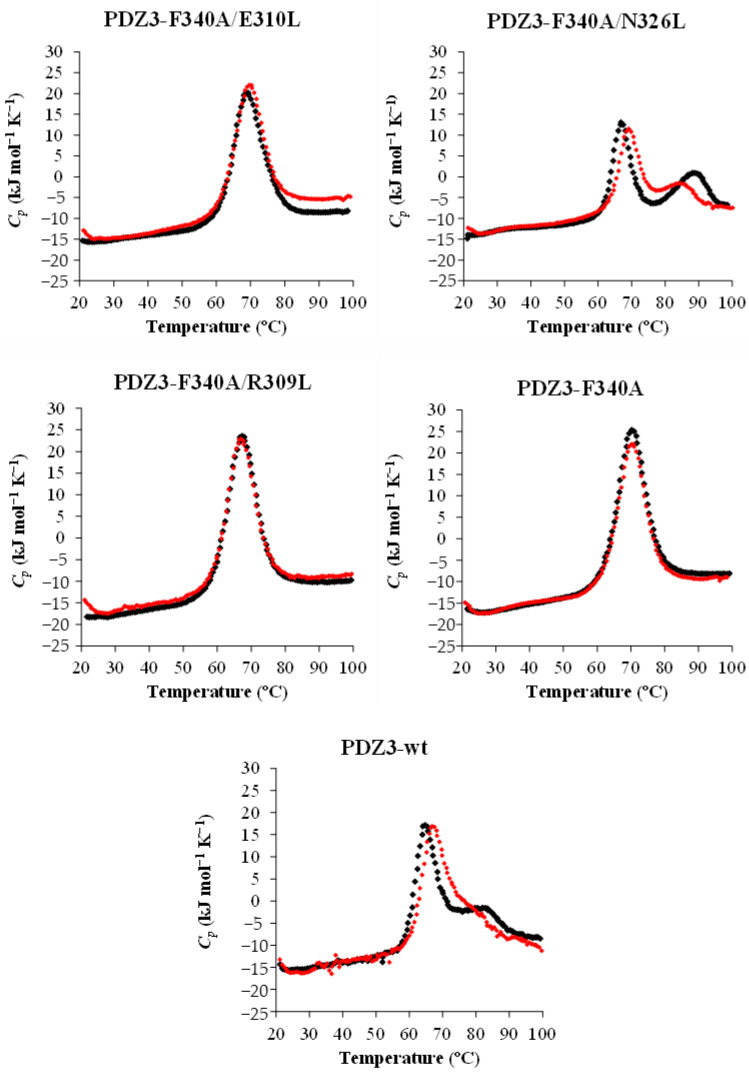
Concentration dependence of DSC thermograms of PDZ3 variants at 0.5–1 mg/mL, pH 7.5, and 1 °C/min scan rate. Black and red dots show DSC thermograms at a protein concentration of 1 and 0.5 mg/mL, respectively.

The DSC thermograms of reversely engineered PDZ3 variants were measured at a 0.5–1 mg/mL concentration in pH 7.5 with a +1 °C/min scan rate. A single endothermic peak was observed in DSC thermograms of PDZ3-F340A, as reported earlier [16], and for PDZ3-F340A/R309L and PDZ3-F340A/E310L. On the other hand, PDZ3-F340A/N326L exhibited two distinct endothermic peaks similar to those observed for PDZ3-wt (Figure 3).

**Figure 4:**
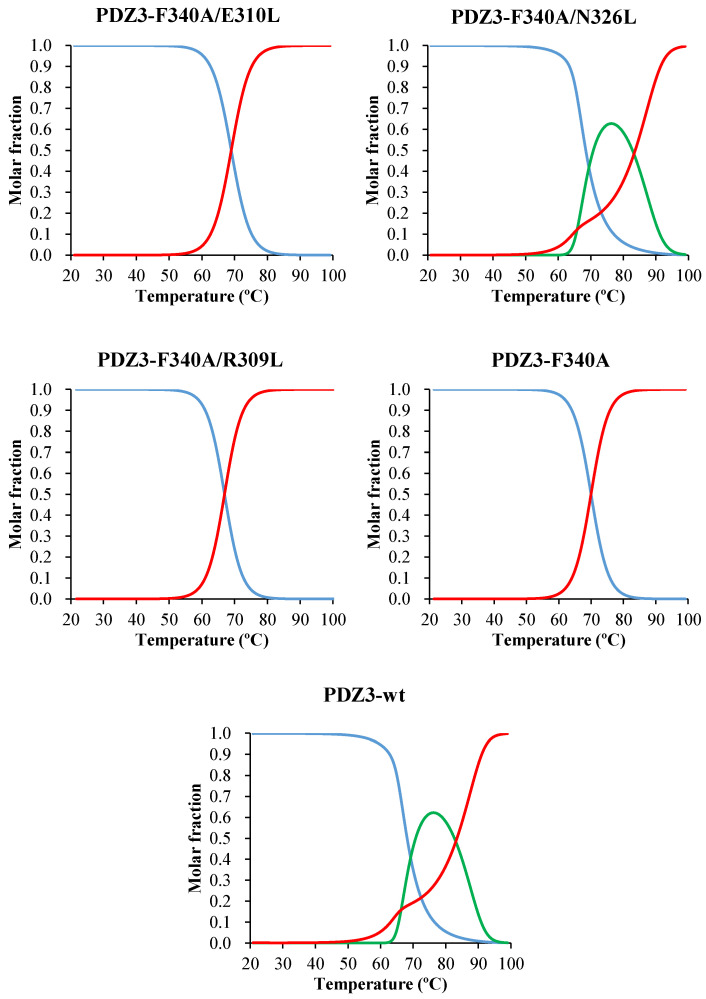
Molar fraction of PDZ3-F340A/E310L (N-D), F340A/N326L (N-I_4_-D), F340A/R309L (N-D), PDZ3-F340A (N-D model) and PDZ3-wt (N-I_5_-D model) by DDCL3 analysis of DSC thermograms at 1 mg/mL, pH 7.5, and 1 °C/min scan rate. The lines represent natively folded monomers (blue), intermediate oligomers (green), and unfolded monomers (red).

**Figure 5:**
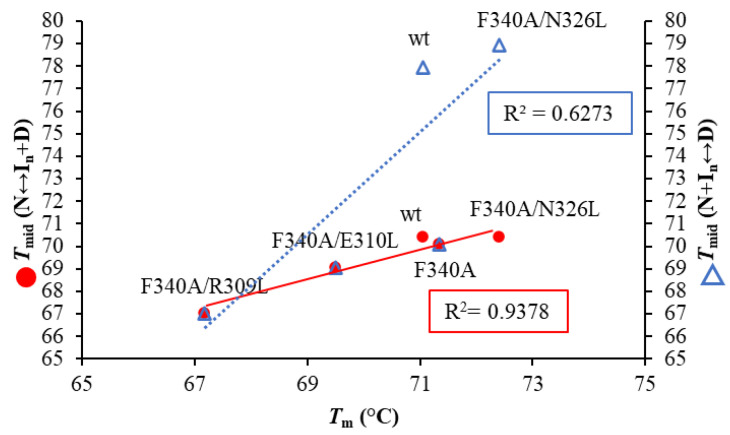
Correlation plots between *T*_m_ calculated by CD denaturation curve and *T*_mid_ (N↔I_n_ + D), *T*_mid_ (N + I_n_↔D) calculated from the molar fractions as determined by DSC analysis. The CD denaturation curve and DSC measurement were measured at 0.5 mg/mL and pH 7.5 at a scan rate of +1.0 °C/min. The red circles (●) represent *T*_mid_ (N↔I_n_+D) and the blue open triangles (∆) present *T*_mid_ (N + I_n_↔D).

A detailed analysis of the DSC curves was performed using DDCL3 with a two- and a three-state model (Table 3, Figure S4). The template PDZ3-F340A, PDZ3-F340A/E310L, and PDZ3-F340A/R309L were well fitted with a two-state model (N↔D). Global fitting of curves with protein concentrations of 0.5–1.0 mg/mL showed that PDZ3-F340A/N326L and PDZ3-wt formed tetrameric and pentameric ROs (N↔1/4(I_4_)↔D and N↔1/5(I_5_)↔D), respectively (Figure 4 and Figure S5). In addition, we observed a strong correlation between *T*_mid_ (N↔ I_n_+D) and the apparent *T*_m_ determined by CD (Figure 5 and Figure S6), but not between *T*_mid_ (N + I_n_↔D) and *T*_m_. This result suggests that the secondary structures of PDZ3-F340A/N326L and PDZ3-wt in the intermediate state is unfolded.

### 2.4. High-Temperature ROs and Amyloidogenesis of the Variants

**Figure 6:**
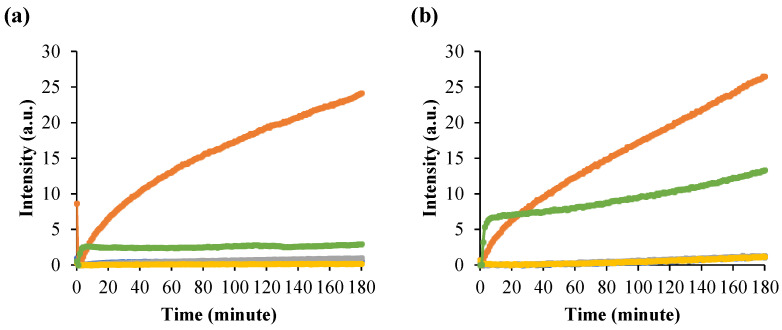
Time-course of RO formation of PDZ3 variants monitored by (**a**) ThT fluorescence at λ_Em_ 480 nm. The dye concentration was 12 μM; (**b**) ANS fluorescence at λ_Em_ 480 nm. The dye concentration was 20 μM. Protein concentration was 1 mg/mL in pH 7.5. Mixture samples were measured at 60 °C for 180 min. PDZ3-F340A/E310L (blue); PDZ3-F340A/N326L (orange); PDZ3-F340A/R309L (gray); PDZ3-F340A (yellow); and PDZ3-wt (green).

To gain insight into the concurrent formation of high-temperature ROs with amyloidogenicity in PDZ3, we monitored the ThT and ANS fluorescence (Figure 6 and Figure S7). We used ThT to monitor amyloidogenesis because there is a strong relationship between beta-cross structures’ formation in fiber and oligomer forms [24,25,26]. ANS indicates molten globule-like properties and binds to partially exposed hydrophobic surfaces and cavities but also to aggregates with molten globule-like properties [24,27,28]. 

The ThT fluorescence intensity of PDZ3-F340A/N326L upon incubation at pH 7.5 and 1mg/mL at 60 °C and 70 °C for 3 h (Figure 6a and Figure S7a) increased within 5 min and became 5 times higher than that of PDZ3-wt. On the other hand, the ThT fluorescence of PDZ3-F340A/E310L, PDZ3-F340A/R309L, and PDZ3-F340A was essentially negligible. Thus, a single mutation, N326L, not only induced RO but strongly increased amyloidogenicity. For the purpose of discussion, let us note that the simultaneous increase of ANS and ThT fluorescence was also observed for Lysozyme [29].

ANS fluorescence of PDZ3-F340A/N326L slightly increased while PDZ3-wt increased within 10 min when incubated at 60 °C and 70 °C at pH 7.5 (Figure 6b and Figure S7b). After 3 h of incubation, the fluorescent intensity of PDZ3-F340A/N326L was 2.5-fold higher than the PDZ3-wt signal. The ANS fluorescence of the other variants was small. A similar phenomenon appeared at 70 °C incubation, but the fluorescence intensity of PDZ3-F340A/N326L and PDZ3-wt rapidly increased at the beginning and with higher intensity than at 60 °C incubation. This result suggests that PDZ3-F340A/N326L in the RO state has a molten globule-like property.

Finally, we measured the hydrodynamic radii (*R*_h_) of the PDZ3 oligomers at temperatures from 25 °C to 90 °C (Figure S8). First, the *R*_h_ of PDZ3 variants were two to three-times larger at high than ambient temperatures (Table 4). In contrast, PDZ3-F340A/N326L showed a hydrodynamic radius (*R*_h_) of 6.74 ± 0.13 nm at 70 °C, and the oligomerization was not fully reversible, as assessed by *R*_h_ measured after cooling the sample down to 25 °C. However, at a protein concentration of 0.5 mg/mL, the oligomerization was fully reversible (Figure S9 and Table S4), strongly suggesting the reversibility of the oligomer formed by N326L mutant, which we defined as a basic property of the RO state (R stands for reversible).

### 2.5. The N326L Mutation Induces RO and Amyloidogenesis

Despite being still rare, the number of high-temperature RO observations is gradually increasing, albeit under non-physiological conditions (Cytochrome c) [30], artificially redesigned protein for controlling solubility (tagged BPTI) [19], or globular domains with natural sequences (Dengue4 envelope domain 3 (DEN4ED3) [31]. In addition, we previously showed that the intermediate state (or RO) could be fully inhibited by replacing a single hydrophobic residue at the crystal interface with an alanine [16,21,31], which correlated with the inhibition of amyloidogenicity.

Here, we used a reversed engineering strategy to assess whether increasing the hydrophobicity of the crystal interface induces RO and concurrent amyloidogenicity. We constructed three variants and confirmed that their structure and physicochemical properties were conserved at low and ambient temperatures (Figures S1 and S6, and Table S5). Our experiment unambiguously indicated that one of the variants, PDZ3-F340A/N326L undergoes a three-state denaturation (N↔ 1/4(I_4_) ↔D), indicating that a single mutation can induce the formation of RO at high-temperature. In contrast, PDZ3-F340A/E310L and PDZ3-F340A/R309L do not induce RO as shown by a single endothermic peak in DSC thermograms and undergo a two-state denaturation (N↔D) like our template PDZ3-F340A.

It remains unclear why the N326L mutation-induced RO, but not R309L nor E310L, despite all three mutations being located at the crystallographic tetramer interface. Further inspection indicated that N326 is located on the β2 strand of PDZ3-F340A, whereas E310 and R309 are in a loop close to the β1-strand. In addition, the aggregation-prone region of PDZ3-F340A calculated by TANGO [32] indicated that N326 is placed in the 323–328 β2 strand, which has a high amyloidogenicity tendency (Figure 1b). On the other hand, R309 and E310 are in nonamyloidogenic regions. These regions were confirmed in PDZ3-wt by heteronuclear NMR experiments, being β2 engaged in the intermediate arrangement of the unfolding intermediate, whereas the β1 region does not [33]. The F340A and L342A mutations, which abolished RO and amyloidogenicity in the PDZ3-wt, were also located in the 335–343 region crossing the β3 strand amyloidogenic regions (data not shown). Thus, although E310 and R309 are located at the interface, which we assumed would increase the hydrophobic interaction and induce RO formation, they did not induce RO nor amyloidogenicity. In addition, the accessible surface area model created by PyMOL showed that the 323–328 region is buried when proteins arrange into the tetramer, but the 335–343 region is not (Figure 1c).

## 3. Materials and Methods

### 3.1. Protein Expression, Purification, and Identification

The proteins were prepared according to our previously reported protocol. In short, single mutations were introduced using a Quikchange protocol and a synthetic gene encoding PDZ3 cloned into a pBAT4 vector as the template. All variants were overexpressed in *Escherichia coli* strain BL21(DE3) with 1 L of LB medium. Protein expression was induced by adding 0.2 mM IPTG when the OD at 590 nm reached 0.6, and the culture was further incubated at 37 °C, 120 rpm for 4 h. The harvested cells were dissolved in 20 mL of 50 mM Tris-HCl (pH 8.7) and lysed by ultrasonication. The supernatant fraction of the cell lysate was then acidified to pH 3 by adding around 1 mL of 1 M HCl and ultracentrifuged. The recombinant proteins were purified from the supernatant by reverse-phase HPLC, lyophilized, and stored at −30 °C until use, as we reported in previous reports [34,35].

The molecular weight of the protein was confirmed by matrix-assisted laser desorption/ionization-time of flight (MALDI-TOF) MS measurements using the plate with Autoflex speed TOF/TOF (Bruker Daltonics, Fremont, CA, USA). The matrix solution was prepared by dissolving 10 mg of sinapic acid in 1 mL of a solution containing 300 μL of acetonitrile, 100 μL of 1% trifluoroacetic acid, and 600 μL of Milli Q water. Protein samples were prepared by mixing 1 μL of protein solution with 9 μL of the matrix solution. 1 μL of 10 µM, 1 µM, and 0.1 µM sample mixtures were spotted and air-dried on the MALDI-TOF MS plate. The molecular weights of all proteins were within 7 Da of the theoretical values (Table S6) computed with ExPASy’s ProtParam tool (https://web.expasy.org/protparam/ accessed on 30 May 2020).

Samples were prepared by dissolving lyophilized proteins in Milli Q water, and the protein concentrations of samples were adjusted to 0.2, 0.5, and 1.0 mg/mL in 50 mM potassium phosphate buffer (pH 7.5). The protein concentrations were determined by measuring absorbance at 280 nm (ε = 2980 M^−1^ cm^−1^) using a Nanodrop (Thermo Fisher Scientific, Waltham, MA, USA). The pH of the samples was confirmed just before performing the experiments. Freshly prepared samples were used for CD, DLS, and fluorescence spectroscopy measurements.

### 3.2. Differential Scanning Calorimetry (DSC) Measurements

Samples were prepared by dissolving lyophilized proteins in Milli Q water and dialyzed for 18 h at 4 °C in 50 mM potassium phosphate buffer (pH 7.5) using a Spectra/Por 3 membrane (MWCO of 3.5 kDa) with one buffer exchange. After dialysis, the protein concentration of samples was adjusted to 1 mg/mL and filtered with Disposable Ultrafiltration Unit, 200 K MWCO (ADVANTEC^®^, Tokyo, Japan), to remove aggregates. Protein concentrations and pHs of the samples were confirmed just before performing experiments.

DSC measurements were performed using a VP-DSC microcalorimeter (Malvern Panalytical Ltd, Malvern, UK) at a scan rate of +1.0 °C/min in the temperature range of 20 to 100 °C, essentially in line with our previous reports [36,37]. Baselines were recorded before measurements using a 50 mM potassium phosphate buffer (pH 7.5). The reversibility of the thermal unfolding was checked by repeating scans of the same sample. Thermodynamic parameters (*T*_mid_ and Δ*H*(*T*_mid_)) were determined by analyzing the apparent heat capacity curves using a non-linear, least-squares fitting algorithm, DDCL3, and assuming a linear temperature dependence of the heat capacity of the native and denatured states [5,38].

### 3.3. Circular Dichroism (CD) Measurements

CD measurements was performed using a Jasco-J820 spectropolarimeter (Tokyo, Japan). A quartz cuvette with 2-mm optical path length was used. The secondary structure contents from CD spectra were calculated using BeStSel [39]. Thermal stability was measured at a protein concentration of 0.5 mg/mL in 50 mM potassium phosphate buffer (pH 7.5), at a +1.0 °C/min scan rate, and monitored between 25 °C and 90 °C using the CD value at 220 nm. Melting temperatures (*T*_m_) were computed through least-squares fittings of experimental data to a two-state model using Origin 2020b (OriginLab Corp, Northampton, MA, USA) [40].

### 3.4. Dynamic Light Scattering (DLS) Measurements

DLS measurements were performed using a glass cuvette with a Zeta-nanosizer (Nano S, Malvern, UK). The sample was measured at 25–90 °C and reversed to 25 °C. The hydrodynamic radius (*R*_h_) was calculated using the Stokes-Einstein equation from size-number plots [41].

### 3.5. Fluorescence Spectroscopy Measurement

ANS fluorescence was measured at an excitation wavelength of 380 nm at 60, 70 °C for 53 h. The emission spectra were monitored from 400 to 600 nm. ThT fluorescence was measured with an excitation wavelength of 444 nm, and the emission spectra were observed from 460 to 640 nm. The final concentration of Thioflavin T (ThT) and 8-Anilino-1-naphthalenesulfonate (ANS) was 12 and 20 μM, respectively. The dye was mixed with 300 μL of the samples, from which 60 μL were transferred to a 3.00 mm Hellma^®^ micro cuvette.

### 3.6. Analytical Ultracentrifugation (AUC) Measurements

Samples were prepared by dissolving lyophilized proteins in Milli Q water and dialyed for 18 h at 4 °C against 50 mM potassium phosphate buffer pH 7.5. The protein concentrations of the samples were adjusted to 1 mg/mL by diluting with the dialyzed buffer. Samples were filtered with a 0.20 μm membrane filter (MilliporeSigma, Burlington, VT, USA) for removing aggregates. Protein concentrations and pH values of the samples were confirmed just before performing the experiments.

Sedimentation velocity experiments were carried out using an Optima XL-I analytical ultracentrifuge (Beckman-Coulter) with An-50 Ti analytical 8-place titanium rotor at 25 °C. Samples were transferred to a 12-mm double-sector epon charcoal-filled centerpiece and centrifuged at a rotor speed of 50,000 rpm, and the absorbance was monitored at 280 nm. Sedimentation velocity data were analyzed using the continuous distribution c(s) analysis module in the SEDFIT software [42]. The range of sedimentation coefficients, where the main peak was present, was integrated to obtain the weighted average sedimentation coefficient. The c(s) distribution was converted into c(M), a molar mass distribution. Solvent density, viscosity, and protein partial specific volumes were calculated using SEDTERP [43].

## 4. Conclusions

The reverse engineering strategy confirmed that hydrophobic interaction at the interface of the monomeric unit in the crystal tetramer induces the RO formation at a high-temperature. Thermodynamic analysis of the DSC denaturation curves indicated that the molar fraction of RO becomes maximal at 60 to 70 °C and that the secondary structures of PDZ3-F340A/N326L and PDZ3-wt in the intermediate state (RO state) are unfolded. Furthermore, the reversed engineering strategy confirmed the relationship between RO appearance and amyloidogenicity. Kinetic experiments need to establish whether ROs are on or off-pathway for amyloidogenesis, but the present results strongly favor the on-pathway hypothesis.

## Figures and Tables

**Table 1:** ASA, BSA, and total RSA of candidate residues of PDZ3-F340A.

Residue	Monomeric ASA (Å^2^)				Tetrameric ASA (Å^2^)				Tetrameric BSA (Å^2^)				Total RSA (%)
	A	B	C	D	A	B	C	D	A	B	C	D	
GLU 310	142	145	140	143	28	145	140	46	114	0	0	97	95
ASN 326	109	107	110	107	109	23	21	107	0	84	89	0	89
ARG 309	216	218	220	218	27	218	220	202	189	0	0	16	75
ASN 369	154	160	157	160	154	17	157	160	0	143	0	0	73
GLU 331	175	175	173	174	175	12	173	174	0	163	0	0	73
THR 321	76	77	76	80	76	20	8	80	0	57	68	0	73
ARG 368	177	150	174	153	84	131	174	74	93	19	0	79	70
LEU 342	85	87	84	85	85	17	17	85	0	70	67	0	68
ALA 343	88	86	91	88	88	47	48	88	0	39	43	0	64
GLY 333	96	100	96	98	96	34	96	98	0	66	0	0	63
SER 320	131	132	132	131	131	100	66	131	0	32	66	0	63
ARG 313	169	169	164	172	90	169	164	81	79	0	0	91	62
PRO 311	67	69	65	67	20	69	65	17	47	0	0	50	61
GLU 395	150	146	148	149	38	146	148	149	112	0	0	0	50
LYS 380	138	138	138	140	138	113	50	140	0	25	88	0	48
GLY 322	25	24	22	25	25	2	0	25	0	22	22	0	42
ILE 389	50	48	49	48	10	48	49	11	40	0	0	37	39
VAL 328	34	35	33	33	34	2	2	33	0	33	31	0	37
ASP 366	108	91	107	88	75	68	107	77	33	23	0	11	35
GLN 391	43	44	43	46	2	44	43	9	41	0	0	37	35

Residues with ASA > 0 Å^2^ of at least one of the chains are listed. The monomeric and tetrameric ASA values of four polypeptide chains (A, B, C, D) of PDZ3-F340A were calculated by DSSP. PDZ3-F340A and other variants were modeled using X-ray crystallographic data of PSD95-PDZ3 (PDB ID: 3I4W) consisting of four monomeric chains in each asymmetric unit cell using COOT (crystallographic object-oriented toolkit) [22] The modeled structures were used for calculating accessible surface area (ASA) by DSSP. Buried surface area (BSA) was calculated by subtracting the ASA in the tetrameric structure from the calculated ASA of the monomeric structure. Relative solvent accessibility (RSA) is the total tetrameric ASA of each residue divided by the maximum amino acid solvent accessibility from theoretical normalization values [23]. The selected residues are underlined.

**Table 2:** Apparent *T*_m_ and van’t Hoff enthalpy (Δ*H*_van’t Hoff_ (*T*_m_)).

Name	*T*_m_ (°C)	Δ*H*_van’t Hoff_ (*T*_m_) (kJ/mol)
PDZ3-F340A/E310L	69.5 ± 0.0	375.6 ± 3.3
PDZ3-F340A/N326L	72.4 ± 0.0	309.5 ± 2.6
PDZ3-F340A/R309L	67.2 ± 0.0	363.1 ± 2.7
PDZ3-F340A	71.3 ± 0.0	367.4 ± 3.6
PDZ3-wt	71.1 ± 0.1	255.3 ± 3.2

The parameters were calculated by fitting the CD denaturation curves using a two-state model in Origin 2020b software. CD denaturation curves were measured at 0.5 mg/mL, pH 7.5, 25–90 °C and +1.0 °C/min scan rate.

**Table 3:** Thermodynamics parameters were calculated by DSC measurements at 0.5–1 mg/mL of protein concentration.

Name	Concentration (mg/mL)	Transition	*T*_mid_ (°C)	Δ*H*_cal_ (*T*_mid_) (kJ/mol)
PDZ3-F340A/E310L	1	N-D	68.9 ± 0.1	341.8 ± 2.7
	0.5	N-D	69.3 ± 0.1	343.1 ± 1.9
PDZ3-F340A/N326L	1	N-I_4_	68.1 ± 0.2	233.4 ± 10.1
		N-D	73.4 ± 1.2	250.8 ± 16.8
	0.5	N-I_4_	70.5 ± 0.1	239.0 ± 7.3
		N-D	72.8 ± 0.7	242.7 ± 4.4
PDZ3-F340A/R309L	1	N-D	67.0 ± 0.1	364.6 ± 2.6
	0.5	N-D	66.8 ± 0.1	359.9 ± 2.7
PDZ3-F340A	1	N-D	70.0 ± 0.1	369.0 ± 2.6
	0.5	N-D	70.2 ± 0.2	358.7 ± 2.7
PDZ3-wt	1	N-I_5_	64.9 ± 0.1	251.4 ± 3.7
		N-D	67.9 ± 0.3	296.0 ± 4.4
	0.5	N-I_5_	67.8 ± 0.1	250.4 ± 8.2
		N-D	69.5 ± 0.5	316.2 ± 8.5

Midpoint temperature (*T*_mid_), calorimetric enthalpy (Δ*H*_cal_ (*T*_mid_)) of PDZ3 variants at pH 7.5 determined by fitting the DSC thermogram using DDCL3. PDZ3-F340A/E310L (N-D model); PDZ3-F340A/N326L (N-I_4_-D model); PDZ3-F340A/R309L (N-D model); PDZ3-F340A (N-D model); PDZ3-wt (N-I_5_-D model).

**Table 4:** Hydrodynamic radius (nm, *R*_h_) by DLS.

Name	25 °C	40 °C	60 °C	70 °C	80 °C	90 °C	25 °C (Reverse)
PDZ3-F340A/E310L	1.76 ± 0.04	1.46 ± 0.24	0.99 ± 0.11	0.94 ± 0.55	1.88 ± 0.02	1.63 ± 0.10	1.58 ± 0.07
PDZ3-F340A/N326L	1.15 ± 0.62	1.60 ± 0.03	0.82 ± 0.06	6.74 ± 0.13	7.94 ± 0.22	7.36 ± 0.98	5.68 ± 0.24
PDZ3-F340A/R309L	1.62 ± 0.03	1.62 ± 0.05	1.70 ± 0.01	2.00 ± 0.05	2.08 ± 0.01	2.08 ± 0.02	1.48 ± 0.02
PDZ3-F340A	1.54 ± 0.04	1.56 ± 0.02	1.66 ± 0.00	1.81 ± 0.07	2.23 ± 0.02	2.41 ± 0.13	1.61 ± 0.02
PDZ3-wt	1.65 ± 0.00	1.46 ± 0.02	1.65 ± 0.03	3.61 ± 0.07	3.06 ± 0.03	2.41 ± 0.05	1.66 ± 0.06

DLS was measured at 1 mg/mL, pH 7.5, and 25–90 °C, and after cooling the sample back to 25 °C after heating. *R*_h_ values were calculated from size-volume graphs. The errors are the standard deviation of three-times measurements with the same sample.

## Data Availability

Data is contained within the article or Supplementary Materials.

## Supplementary Materials

The following supporting information can be downloaded at: https://www.mdpi.com/article/10.3390/molecules27092813/s1, Figure S1: Accessible surface models of PDZ3 variants and PDZ3-wt by Pymol; Figure S2: Sedimentation velocity analysis of PDZ3 variants by AUC measurements; Figure S3: CD spectra of PDZ3 variants; Figure S4: Concentration dependence of DSC thermograms of PDZ3 variants fitting by DDCL3 analysis; Figure S5: Molar fraction of PDZ3 variants calculated by DDCL3 analysis of DSC thermograms; Figure S6: Fitting curve calculated by CD thermal denaturation superimposed to the molar fraction of I_n_ + D state calculated from DSC thermograms; Figure S7: Time-course fluorescence spectra of PDZ3 variants at 70°C by monitoring ThT and ANS fluorescence; Figure S8: Hydrodynamic radii (*R*_h_) of PDZ3 variants using DLS measurements; Figure S9: Bar plot of Hydrodynamic radius (nm, *R*_h_) by DLS measurements; Table S1: Accessible Surface Area values of artificial crystallographic PDZ3-F340A calculated by DSSP; Table S2: Molecular weight of PDZ3 variants determined by MALDI-TOF MS; Table S3: Secondary structure contents of PDZ3 variants calculated by BeStSel; Table S4: Sedimentation velocity analysis of PDZ3 variants at 25°C analyzed using SEDFIT and SEDNTERP; Table S5: Residues of PDZ3 variants between DSC raw data and fitting curves calculated with DDCL3; Table S6: Hydrodynamic radius (nm, *R*_h_) by DLS.

## Sample Availability

The PDZ3-wt plasmid is available from JCM.
