# Supplementary material for: Reverse Engineering Analysis of the High-Temperature Reversible Oligomerization and Amyloidogenicity of PSD95-PDZ3
Source: Molecules. 2022 Apr 28;27(9):2813. doi: 10.3390/molecules27092813 (PMC9103278; doi:10.3390/molecules27092813)
Supplement: Supplementary file 1 [file molecules-27-02813-s001.zip › molecules-1657965-supplementary.pdf]

Supplementary Materials

Figure S1: Accessible surface models of PDZ3 variants and PDZ3-wt by Pymol

Figure S2: Sedimentation velocity analysis of PDZ3 variants by AUC measurements

Figure S3: CD spectra of PDZ3 variants

Figure S4: Concentration dependence of DSC thermograms of PDZ3 variants fitting by DDCL3 analysis

Figure S5: Molar fraction of PDZ3 variants calculated by DDCL3 analysis of DSC thermograms

Figure S6: Fitting curve calculated by CD thermal denaturation superimposed to the molar fraction of I_n+D state calculated from DSC thermograms

Figure S7: Time-course fluorescence spectra of PDZ3 variants at 70°C by monitoring ThT and ANS fluorescence

Figure S8: Hydrodynamic radii (R_h) of PDZ3 variants using DLS measurements

Figure S9: Bar plot of Hydrodynamic radius (nm, R_h) by DLS measurements

Table S1: Accessible Surface Area values of artificial crystallographic PDZ3-F340A calculated by DSSP

Table S2: Molecular weight of PDZ3 variants determined by MALDI-TOF MS

Table S3: Secondary structure contents of PDZ3 variants calculated by BeStSel

Table S4: Sedimentation velocity analysis of PDZ3 variants at 25ºC analyzed using SEDFIT and SEDNTERP

Table S5: Residues of PDZ3 variants between DSC raw data and fitting curves calculated with DDCL3

Table S6: Hydrodynamic radius (nm, R_h) by DLS

PDZ3-F340A/E310L

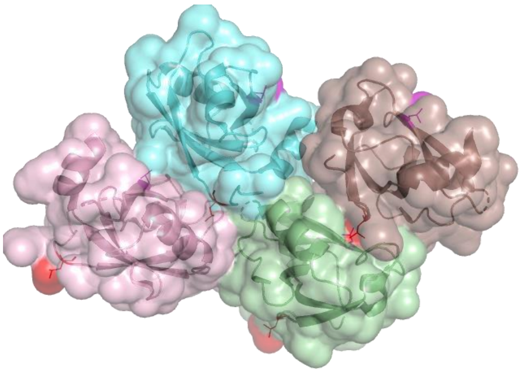

PDZ3-F340A/N326L

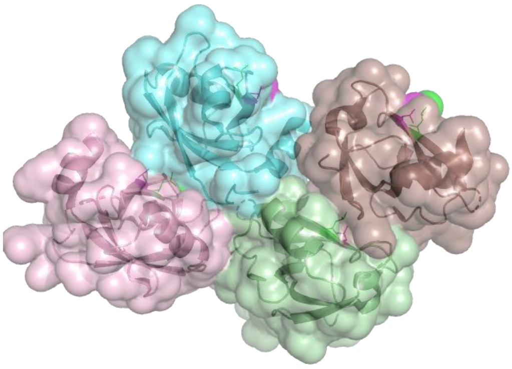

PDZ3-F340A/R309L

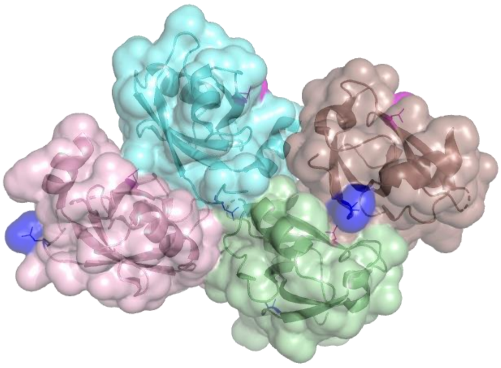

PDZ3-F340A

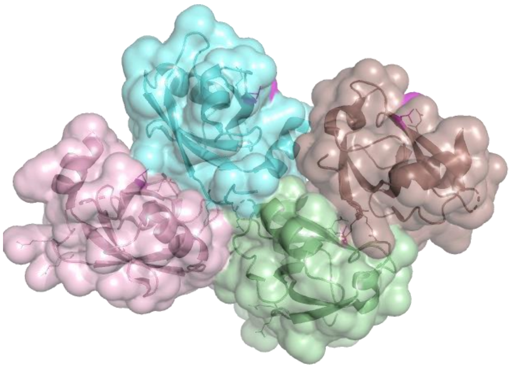

PDZ3-wt

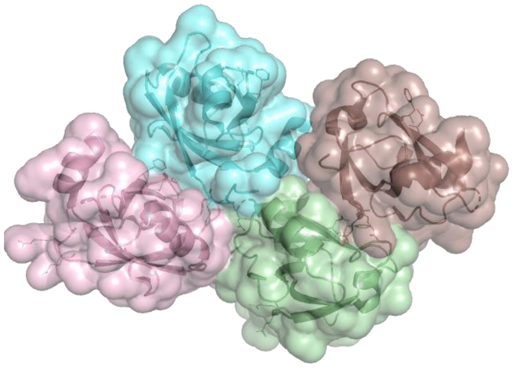

Figure S1: Accessible surface models of PDZ3 variants and PDZ3-wt (PDB ID: 3I4W) drawn using Pymol. The sites of single alanine mutation in PDZ3-F340A template protein are shown in magenta. E310L, N326L, R309L, and F340A are shown in red, green, blue, and magenta. Chains A, B, C, and D are shown in light blue, light green, light pink, and brown.

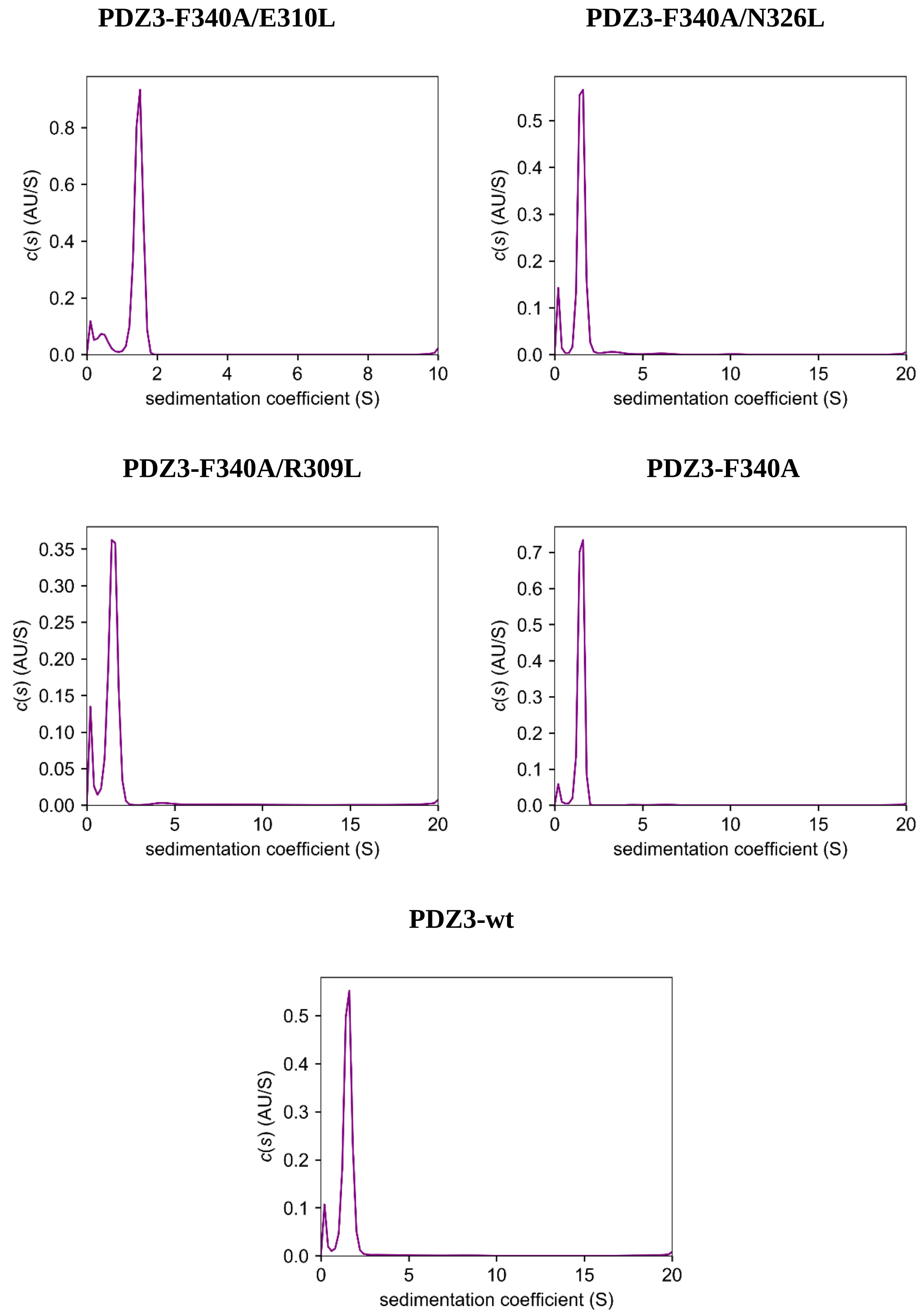

Figure S2: Sedimentation velocity analysis of PDZ3 variants was performed with AUC experiments at 1 mg/mL in 50 mM potassium phosphate buffer pH 7.5 and 25ºC. Samples were centrifuged at a speed of 50,000 rpm, and sedimentation velocity data were analyzed using SEDFIT and SEDNTERP [42-43].

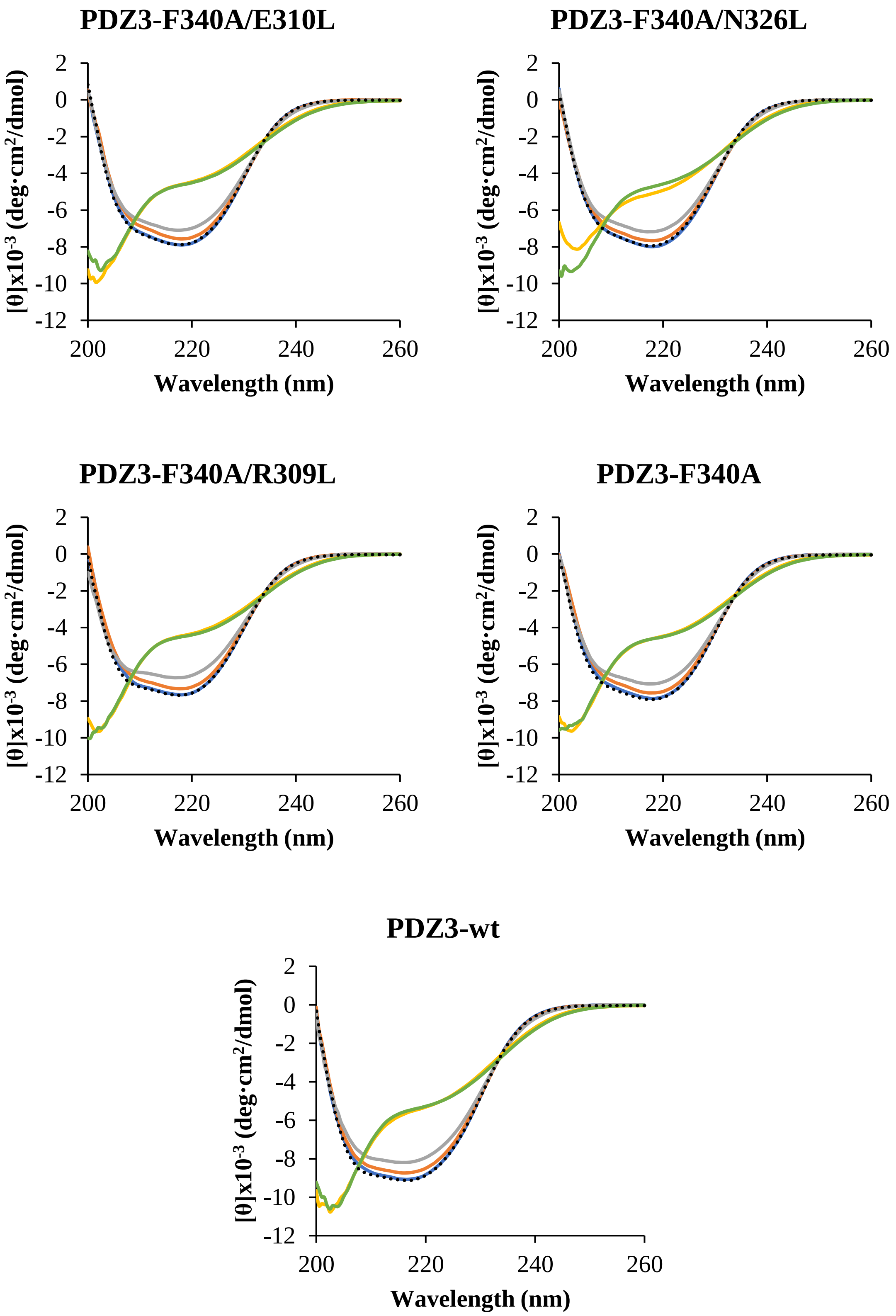

Figure S3: CD spectra of PDZ3 variants at 0.2 mg/mL, pH 7.5 and 25-90°C. Sample were heated up from 25°C to 90°C and cooled down to 25°C. 25°C (blue); 40°C (orange); 60°C (gray); 80°C (yellow); 90°C (green); 25°C after heating (dotted black).

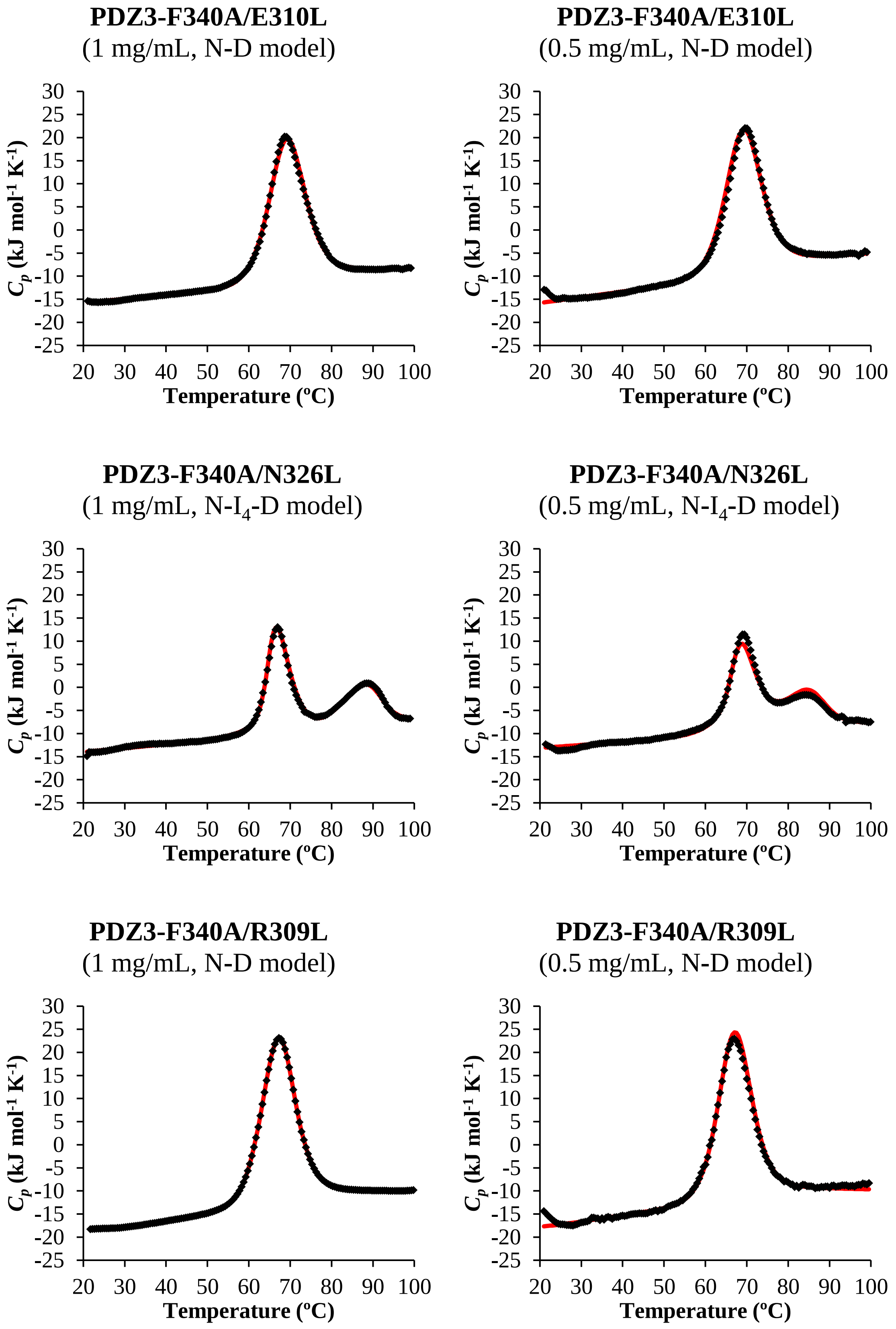
PDZ3-F340A/E310L
(1 mg/mL, N-D model)
PDZ3-F340A/E310L
(0.5 mg/mL, N-D model)
PDZ3-F340A/N326L
(1 mg/mL, N-I4-D model)
PDZ3-F340A/N326L
(0.5 mg/mL, N-I4-D model)
PDZ3-F340A/R309L
(1 mg/mL, N-D model)
PDZ3-F340A/R309L
(0.5 mg/mL, N-D model)
Cp (kJ mol-1 K-1)
Temperature (ºC)
30
25
20
15
10
5
0
-5
-10
-15
-20
-25
20
30
40
50
60
70
80
90
100

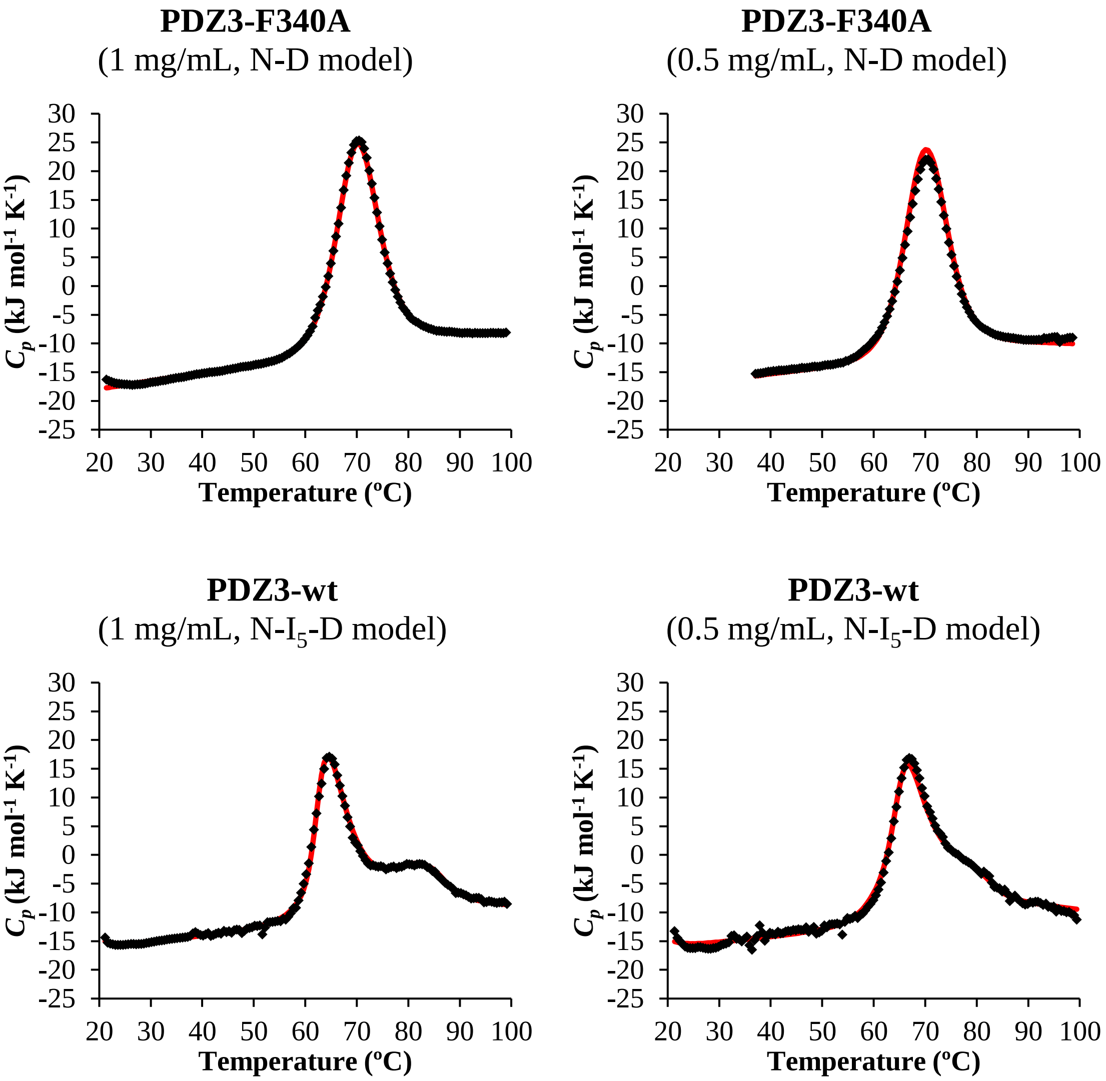

Figure S4: Fitting of PDZ3-F340A/E310L (N-D model), PDZ3-F340A/N326L (N-I_4-D), PDZ3-F340A/R309L (N-D model), PDZ3-F340A (N-D model), and PDZ3-wt (N-I_5-D model) by DDCL3 analysis of DSC thermograms at 0.5 and 1 mg/mL, pH 7.5 and +1°C/min scan rate. Black dot represents DSC raw data at 1 mg/mL and 0.5 mg/mL, respectively. The red line represents the fitting curve.

PDZ3-F340A/E310L

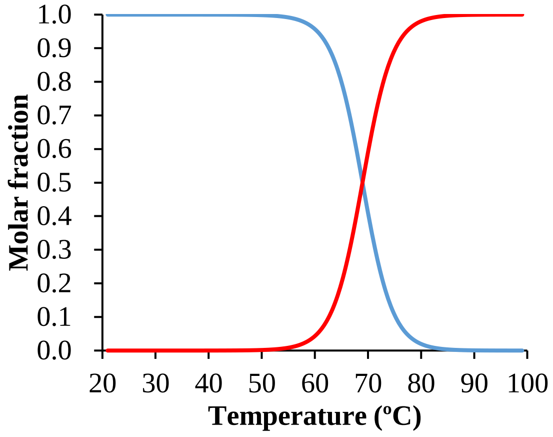

PDZ3-F340A/N326L

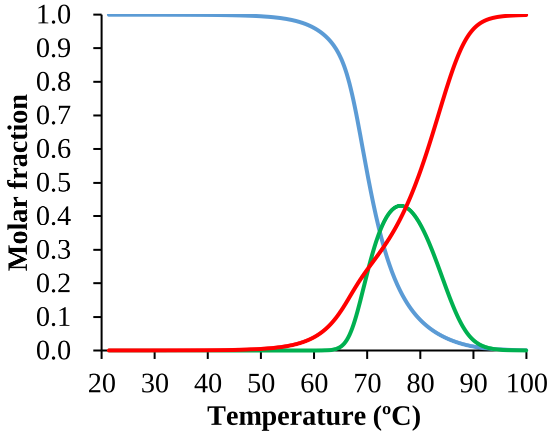

PDZ3-F340A/R309L

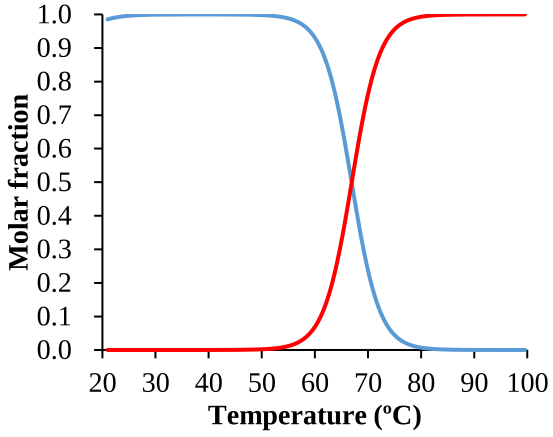

PDZ3-F340A

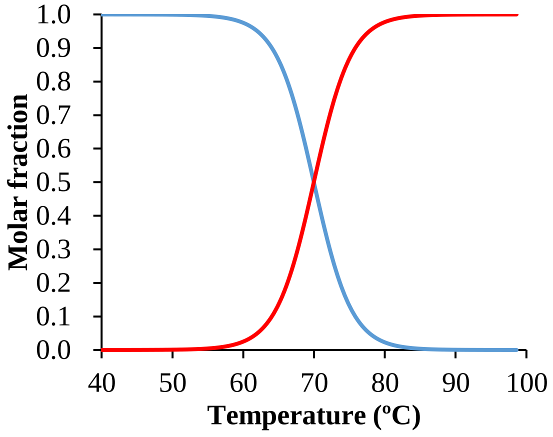

PDZ3-wt

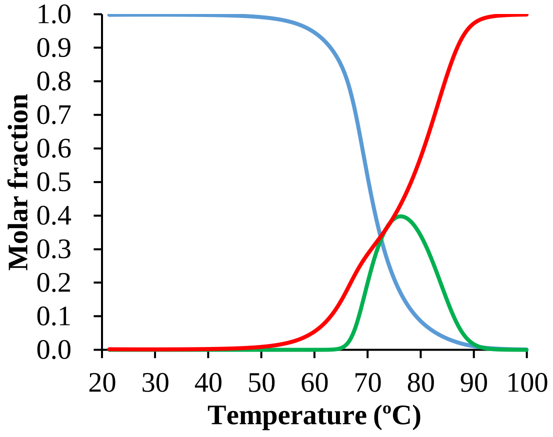

Figure S5: Molar fraction of PDZ3-F340A/E310L (N-D model), PDZ3-F340A/N326L (N-I_4-D model), PDZ3-F340A/R309L (N-D model), PDZ3-F340A (N-D model), PDZ3-wt (N-I_5-D model) by DDCL3 analysis of DSC thermograms at 0.5 mg/mL, pH 7.5 and +1°C/min scan rate. Natively folded monomer (blue); Intermediate oligomer (green); Unfolded monomer (red).

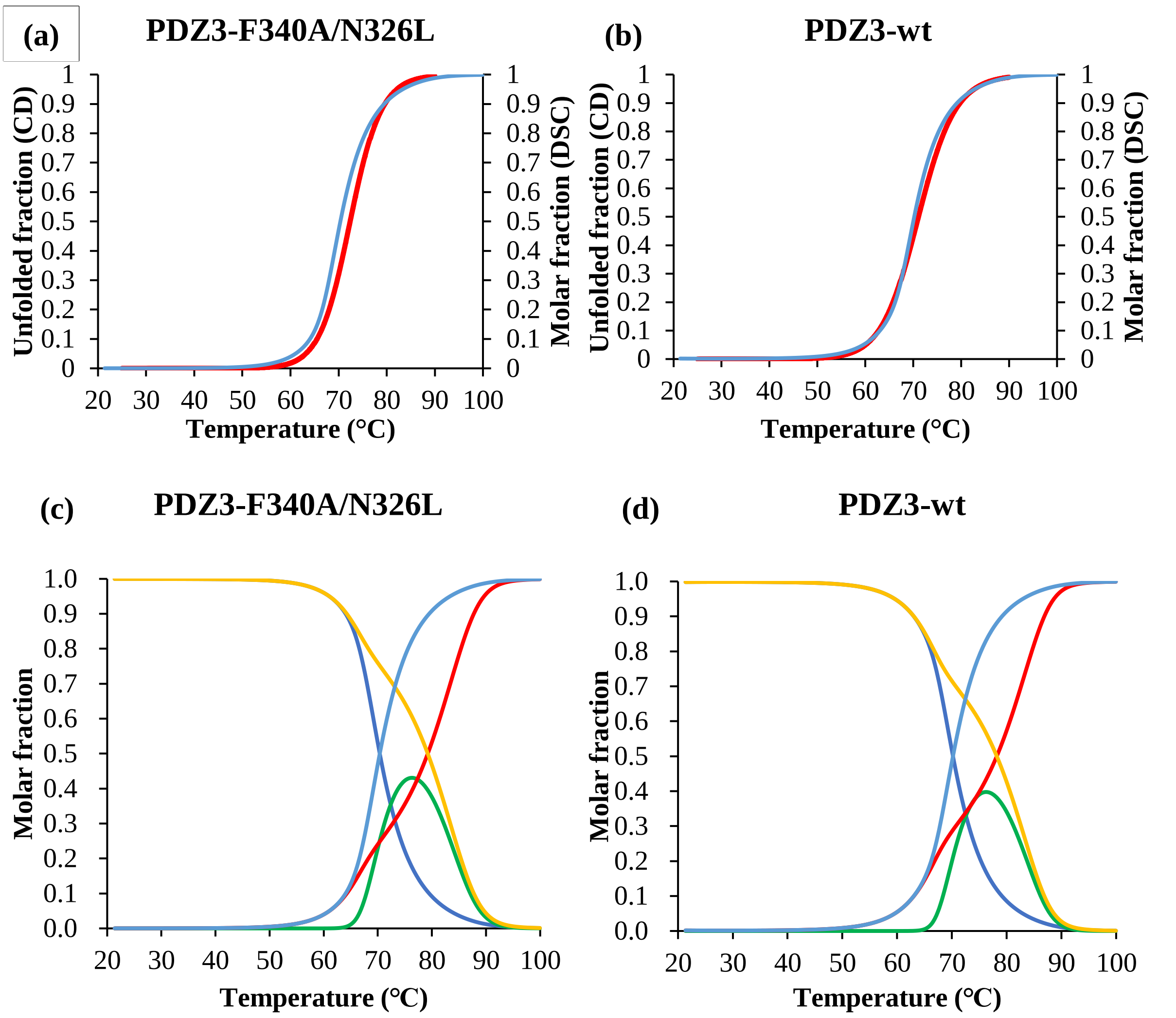

Figure S6: Fitting curve calculated from CD thermal denaturation and molar fraction in the I_n+D state calculated from the DSC thermograms of (a) PDZ3-F340A/N326L; (b) PDZ3-wt. The blue line represents the molar fraction of the I_n+D state, and the red line represents the fitting curve of the CD data. Molar fraction of (c) PDZ3-F340A/N326L (N-I_4-D model); (d) PDZ3-wt (N-I_5-D model) calculated using the DDCL3 analysis of DSC thermograms. Natively folded monomer (N) (dark blue); Intermediate oligomer (I_n) (green); Unfolded monomer (D) (red); Natively folded and intermediate oligomer (N+I_n) (yellow); Intermediate oligomer and unfolded monomer (I_n+D) (light blue). Experiments (CD and DSC) were performed at 0.5 mg/mL and pH 7.5, +1.0 °C/min scan rate.

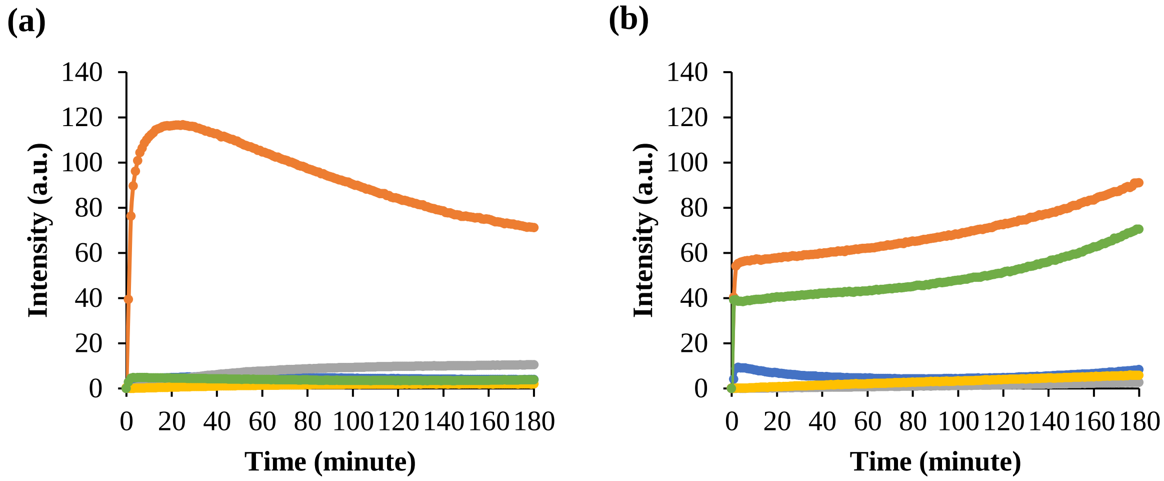

Figure S7: Time-course of RO formation of PDZ3 variants monitored by (a) ThT fluorescence at λ_{Em} 480 nm. The dye concentration was 12 µM; (b) ANS fluorescence at λ_{Em} 480 nm. The dye concentration was 20 µM. Protein concentration was 1 mg/mL in pH 7.5. Mixture samples were measured at 60°C for 180 minutes. PDZ3-F340A/E310L (blue); PDZ3-F340A/N326L (orange); PDZ3-F340A/R309L (gray); PDZ3-F340A (yellow); and PDZ3-wt (green).

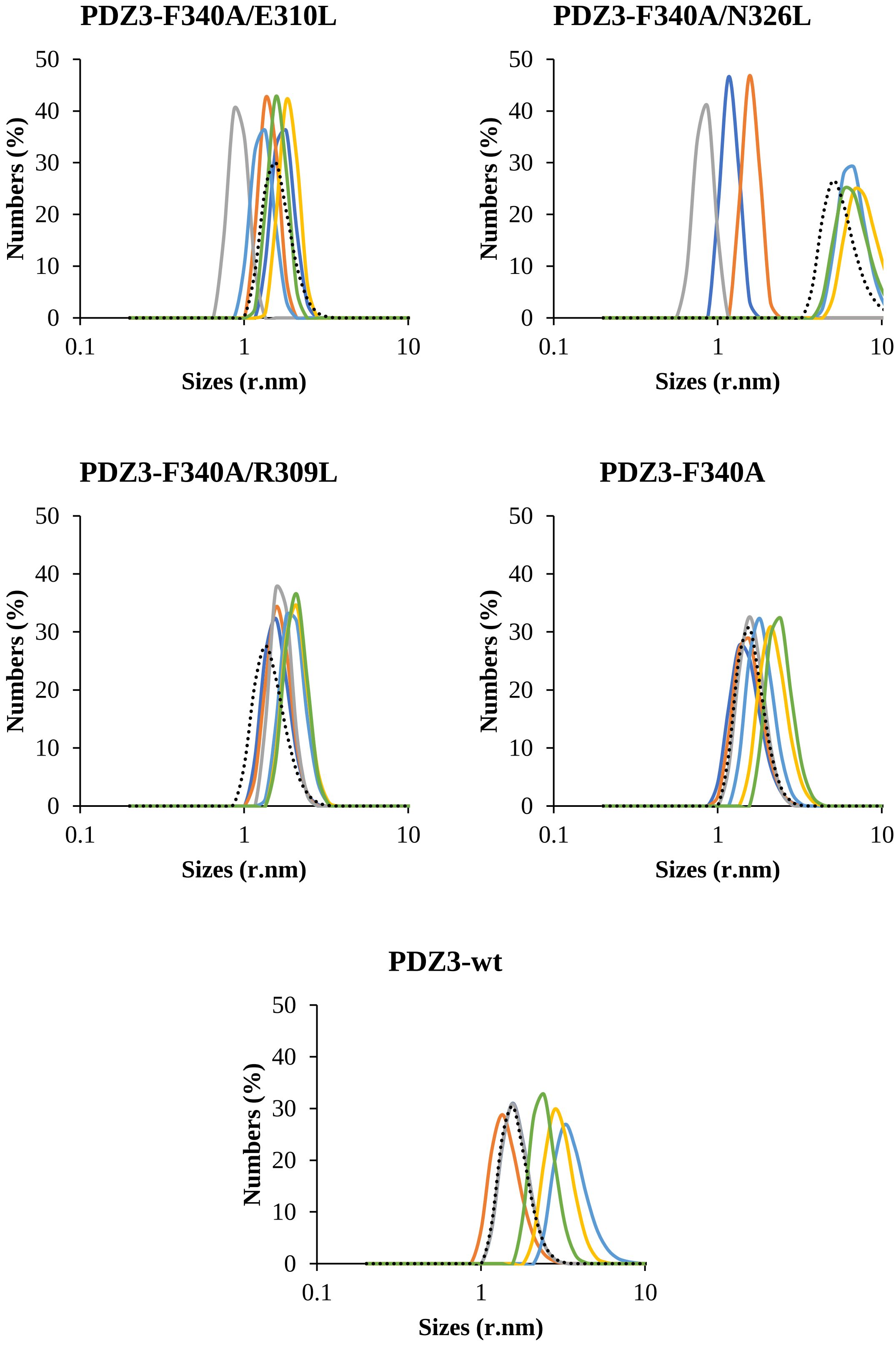

Figure S8: Size-numbers plots of PDZ3 variants at 1 mg/mL, pH 7.5 and 25-90°C by DLS measurements. Hydrodynamic radii (R_h) were measured by increasing the temperature from

25°C to 90°C and decreasing to 25°C. 25°C (blue); 40°C (orange); 60°C (gray); 70°C (cyan); 80°C (yellow); 90°C (green); 25°C after heating (dotted black).

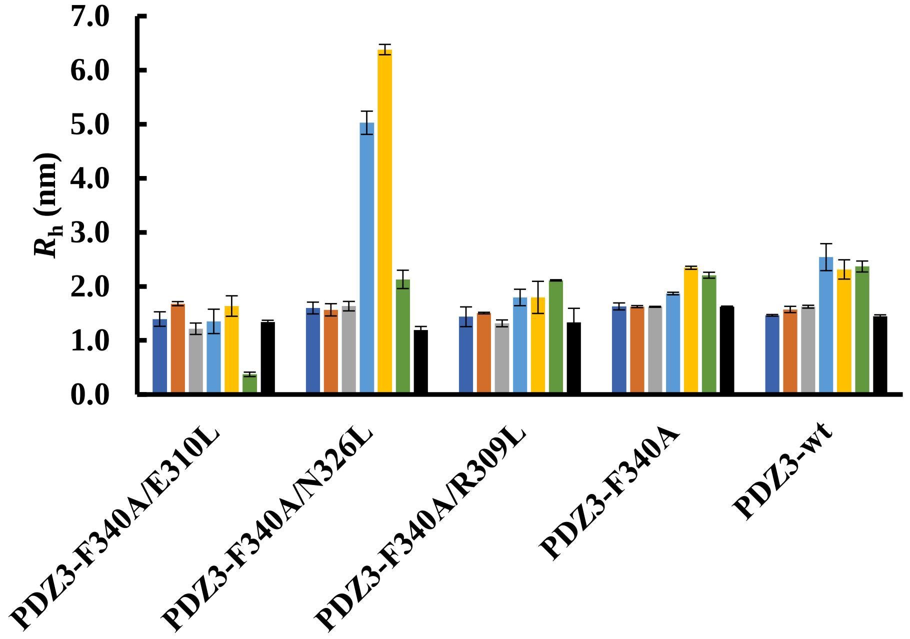

Figure S9: Bar plot of Hydrodynamic radius (nm, R_h) by DLS measurements. Hydrodynamic radii (R_h) of PDZ3 variants at 0.5 mg/mL, pH 7.5 were measured by increasing the temperature from 25°C to 90°C and cooling to 25°C. 25°C (blue); 40°C (orange); 60°C (gray); 70°C (cyan); 80°C (yellow); 90°C (green); 25°C after heating (black).

Table S1: Accessible Surface Area values of artificial crystallographic PDZ3-F340A calculated by DSSP

Residue	Monomeric ASA (Å^2)				Tetrameric ASA (Å^2)				Tetrameric BSA (Å^2)				Total RSA (%)
	A	B	C	D	A	B	C	D	A	B	C	D	
GLU 305	195	194	153	180	124	194	153	180	71	0	0	0	32
ASP 306	135	149	147	131	129	149	147	131	6	0	0	0	3
ILE 307	32	39	22	40	7	39	22	40	25	0	0	0	13
PRO 308	64	71	64	65	35	71	64	54	29	0	0	11	25
ARG 309	216	218	220	218	27	218	220	202	189	0	0	16	75
GLU 310	142	145	140	143	28	145	140	46	114	0	0	97	95
PRO 311	67	69	65	67	20	69	65	17	47	0	0	50	61
ARG 312	36	33	35	30	31	33	35	24	5	0	0	6	4
ARG 313	169	169	164	172	90	169	164	81	79	0	0	91	62
ILE 314	1	1	1	3	1	1	1	3	0	0	0	0	0
VAL 315	50	52	50	53	50	52	50	50	0	0	0	3	2
ILE 316	3	3	3	4	3	3	3	4	0	0	0	0	0
HIS 317	115	114	117	115	115	114	117	75	0	0	0	40	18
ARG 318	55	50	77	51	55	28	45	51	0	22	32	0	20
GLY 319	38	42	38	41	38	37	33	41	0	5	5	0	10
SER 320	131	132	132	131	131	100	66	131	0	32	66	0	63
THR 321	76	77	76	80	76	20	8	80	0	57	68	0	73
GLY 322	25	24	22	25	25	2	0	25	0	22	22	0	42
LEU 323	13	13	12	14	13	8	12	14	0	5	0	0	2
GLY 324	3	4	4	3	3	3	4	3	0	1	0	0	1
PHE 325	19	21	20	21	19	21	20	21	0	0	0	0	0
ASN 326	109	107	110	107	109	23	21	107	0	84	89	0	89
ILE 327	21	23	19	23	21	21	12	23	0	2	7	0	5
VAL 328	34	35	33	33	34	2	2	33	0	33	31	0	37
GLY 329	8	8	8	9	8	0	8	9	0	8	0	0	8
GLY 330	13	14	13	14	13	4	13	14	0	10	0	0	10
GLU 331	175	175	173	174	175	12	173	174	0	163	0	0	73
SNN 332	0	0	0	0	0	0	0	0	0	0	0	0	0
GLY 333	96	100	96	98	96	34	96	98	0	66	0	0	63
GLU 334	19	20	16	18	19	6	16	18	0	14	0	0	6

Residue	Monomeric ASA (Å^2)				Tetrameric ASA (Å^2)				Tetrameric BSA (Å^2)				Total RSA (%)
	**A**	**B**	**C**	**D**	**A**	**B**	**C**	**D**	**A**	**B**	**C**	**D**	
GLY 335	10	10	10	11	10	3	10	11	0	7	0	0	7
ILE 336	0	0	0	0	0	0	0	0	0	0	0	0	0
PHE 337	0	0	0	0	0	0	0	0	0	0	0	0	0
ILE 338	0	0	0	0	0	0	0	0	0	0	0	0	0
SER 339	28	24	27	25	28	5	17	25	0	19	10	0	19
**ALA 340**	43	41	43	41	43	25	31	41	0	16	12	0	12
ILE 341	53	48	52	48	53	48	52	48	0	0	0	0	0
LEU 342	85	87	84	85	85	17	17	85	0	70	67	0	<u>68</u>
ALA 343	88	86	91	88	88	47	48	88	0	39	43	0	<u>64</u>
GLY 344	56	52	50	52	56	50	41	52	0	2	9	0	11
GLY 345	6	4	6	3	6	3	3	3	0	1	3	0	4
PRO 346	31	29	28	31	31	29	28	31	0	0	0	0	0
ALA 347	1	0	0	0	1	0	0	0	0	0	0	0	0
ASP 348	75	68	73	70	75	68	73	70	0	0	0	0	0
LEU 349	143	127	136	127	143	127	136	127	0	0	0	0	0
SER 350	38	28	38	34	38	28	38	34	0	0	0	0	0
GLY 351	64	64	60	61	64	64	60	61	0	0	0	0	0
GLU 352	91	89	89	102	91	89	89	102	0	0	0	0	0
LEU 353	11	11	11	11	11	11	11	11	0	0	0	0	0
ARG 354	71	52	75	68	69	52	75	68	2	0	0	0	1
LYS 355	123	120	124	122	123	120	124	122	0	0	0	0	0
GLY 356	1	1	0	0	1	1	0	0	0	0	0	0	0
ASP 357	0	0	0	0	0	0	0	0	0	0	0	0	0
GLN 358	14	13	14	12	14	13	14	12	0	0	0	0	0
ILE 359	0	0	0	0	0	0	0	0	0	0	0	0	0
LEU 360	23	19	20	20	3	19	20	2	20	0	0	18	19
SER 361	23	23	24	23	15	23	24	18	8	0	0	5	8
VAL 362	1	1	1	1	1	1	1	1	0	0	0	0	0
ASN 363	68	72	69	69	68	72	69	69	0	0	0	0	0
GLY 364	71	68	69	69	71	68	69	69	0	0	0	0	0
VAL 365	74	75	75	76	74	75	75	76	0	0	0	0	0
ASP 366	108	91	107	88	75	68	107	77	33	23	0	11	<u>35</u>

Residue	Monomeric ASA (Å^2)				Tetrameric ASA (Å^2)				Tetrameric BSA (Å^2)				Total RSA (%)
	A	B	C	D	A	B	C	D	A	B	C	D	
LEU 367	3	3	4	3	3	3	4	3	0	0	0	0	0
ARG 368	177	150	174	153	84	131	174	74	93	19	0	79	70
ASN 369	154	160	157	160	154	17	157	160	0	143	0	0	73
ALA 370	12	14	11	13	12	7	11	13	0	7	0	0	5
SER 371	46	44	47	45	46	5	47	45	0	39	0	0	25
HIS 372	73	70	75	71	73	50	71	71	0	20	4	0	11
GLU 373	126	123	108	120	126	91	108	120	0	32	0	0	14
GLN 374	117	118	116	119	117	46	116	119	0	72	0	0	32
ALA 375	0	0	0	0	0	0	0	0	0	0	0	0	0
ALA 376	33	31	32	30	33	31	32	30	0	0	0	0	0
ILE 377	73	75	71	73	73	63	71	73	0	12	0	0	6
ALA 378	14	24	17	23	14	24	17	23	0	0	0	0	0
LEU 379	16	14	14	14	16	12	14	14	0	2	0	0	1
LYS 380	138	138	138	140	138	113	50	140	0	25	88	0	48
ASN 381	111	116	112	115	111	116	88	115	0	0	24	0	12
ALA 382	18	20	17	17	18	20	17	17	0	0	0	0	0
GLY 383	33	35	37	35	33	35	33	35	0	0	4	0	4
GLN 384	133	126	165	127	133	126	156	127	0	0	9	0	4
THR 385	69	71	70	71	69	71	70	71	0	0	0	0	0
VAL 386	0	0	0	0	0	0	0	0	0	0	0	0	0
THR 387	42	39	42	40	42	39	42	40	0	0	0	0	0
ILE 388	0	0	0	0	0	0	0	0	0	0	0	0	0
ILE 389	50	48	49	48	10	48	49	11	40	0	0	37	39
ALA 390	0	0	0	0	0	0	0	0	0	0	0	0	0
GLN 391	43	44	43	46	2	44	43	9	41	0	0	37	35
TYR 392	58	59	47	60	43	59	47	60	15	0	0	0	6
LYS 393	55	55	54	54	32	53	54	53	23	2	0	1	11
PRO 394	49	45	48	47	34	45	48	47	15	0	0	0	9
GLU 395	150	146	148	149	38	146	148	149	112	0	0	0	50
GLU 396	56	67	56	62	46	67	56	62	10	0	0	0	4
TYR 397	28	26	27	25	28	22	27	25	0	4	0	0	2
SER 398	64	62	64	58	63	62	64	58	1	0	0	0	1

Residue	Monomeric ASA (Å^2)				Tetrameric ASA (Å^2)				Tetrameric BSA (Å^2)				Total RSA (%)
	A	B	C	D	A	B	C	D	A	B	C	D	
ARG 399	188	187	187	183	115	187	187	183	73	0	0	0	27
PHE 400	90	92	89	89	90	73	89	89	0	19	0	0	8
GLU 401	71	75	73	73	71	35	69	73	0	40	4	0	20
ALA 402	125	120	124	113	95	120	124	113	30	0	0	0	23

Monomeric ASAs (Accessible Surface Area) and Tetrameric ASAs values of all residues of PDZ3-F340A of the crystallographic tetramer (Chain A, Chain B, Chain C, and Chain D) calculated by DSSP. BSA (Buried Surface Area) obtained by subtraction of tetrameric ASAs (Accessible Surface Area) from Monomeric ASAs. The total relative accessible surface area was calculated as RSA=ASA/Maximum ASA, where maximum ASA was from theoretical normalization values [23].

Table S2: Molecular weight of PDZ3 variants determined by MALDI-TOF MS

Name	Exp. Mw (Da)	Cal. Mw (Da)	Relative error (%)
PDZ3-F340A/E310L	10904.52	10911.29	6.20×10^{-2}
PDZ3-F340A/N326L	10918.97	10926.30	6.71×10^{-2}
PDZ3-F340A/R309L	10886.40	10884.22	2.00×10^{-2}
PDZ3-F340A	10929.82	10927.25	2.35×10^{-2}
PDZ3-wt	11004.68	11003.35	1.21×10^{-2}

Experimental molecular weight (Exp. Mw) determined by MALDI-TOF MS measurements and calculated molecular weight (Cal. Mw) by Protparam (http://web.expasy.org/protparam/). Relative errors are in percentages (|Exp. Mw - Cal. Mw| / Cal. Mw ×100.)

Table S3: Secondary structure contents of PDZ3 variants calculated by BeStSel.

	Helix	Anti-parallel	Parallel	Turn	Others
PDZ3-F340A/E310L	14.0	30.1	3.6	12.0	40.4
PDZ3-F340A/N326L	14.0	29.9	3.2	11.8	41.1
PDZ3-F340A/R309L	12.2	28.8	3.2	12.5	43.3
PDZ3-F340A	12.8	29.3	3.9	12.2	41.7
PDZ3-wt	16.3	25.7	4.9	11.6	41.5

Secondary structure contents of PDZ3 variants calculated by BeStSel (https://bestsel.elte.hu/index.php) [39]. CD spectra of PDZ3 variants were measured at 0.2 mg/mL, pH 7.5 and 25°C.

Table S4: Sedimentation velocity analysis of PDZ3 variants at 25ºC analyzed using SEDFIT and SEDNTERP

Variants	Weighted average sedimentation coefficient (S)	Calculated molecular weight (M_{exp}, Da)	Monomeric population (%)	Calculated molecular weight from amino acid sequence (M_{calc}, Da)	M_{exp}/M_{calc}
PDZ3-F340A/E310L	1.47	9596.74	85.40	10911.3	0.88
PDZ3-F340A/N326L	1.53	10066.47	84.61	10926.3	0.92
PDZ3-F340A/R309L	1.48	9863.68	76.09	10884.2	0.91
PDZ3-F340A	1.50	9775.70	89.01	10927.3	0.89
PDZ3-wt	1.53	10132.56	85.62	11003.4	0.92

Sedimentation velocity analysis of 1 mg/mL, pH 7.5 of PDZ3 variants was performed at 25ºC, and experimental data were analyzed using SEDFIT and SEDNTERP [42-43].

Table S5: Residues of PDZ3 variants between DSC raw data and fitting curves calculated with DDCL3

Name	Model	Residuals (μW)
PDZ3-F340A/E310L	**N-D**	**0.275**
	N-1/3(I_3)-D	0.187
	N-1/4(I_4)-D	0.181
	N-1/5(I_5)-D	0.182
PDZ3-F340A/N326L	N-D	N.A.
	N-1/3(I_3)-D	0.317
	**N-1/4(I_4)-D**	**0.259**
	N-1/5(I_5)-D	0.274
PDZ3-F340A/R309L	**N-D**	**0.253**
	N-1/3(I_3)-D	0.177
	N-1/4(I_4)-D	0.231
	N-1/5(I_5)-D	0.231
PDZ3-F340A	**N-D**	**0.254**
	N-1/3(I_3)-D	0.215
	N-1/4(I_4)-D	0.215
	N-1/5(I_5)-D	0.215
PDZ3-wt	N-D	N.A.
	N-1/3(I_3)-D	0.333
	N-1/4(I_4)-D	0.312
	**N-1/5(I_5)-D**	**0.307**

The oligomer number of the intermediate (I) state varied from trimer (I_3) to pentamer (I_5) at the thermal transition models. Optimal thermal transition models of each PDZ3 variant were determined by comparing the minimal residuals.

Table S6: Hydrodynamic radius (nm, R_h) by DLS

Name	25°C	40°C	60°C	70°C	80°C	90°C	25°C (Reverse)
PDZ3-F340A/E310L	1.40±0.13	1.68±0.04	1.22±0.11	1.35±0.23	1.64±0.19	0.37±0.04	1.34±0.03
PDZ3-F340A/N326L	1.60±0.11	1.57±0.11	1.63±0.09	5.03±0.22	6.38±0.10	2.13±0.17	1.19±0.06
PDZ3-F340A/R309L	1.44±0.18	1.51±0.01	1.32±0.06	1.80±0.15	1.80±0.30	2.11±0.01	1.33±0.26
PDZ3-F340A	1.63±0.06	1.63±0.02	1.62±0.01	1.87±0.02	2.34±0.03	2.21±0.05	1.63±0.01
PDZ3-wt	1.46±0.02	1.57±0.06	1.62±0.03	2.54±0.25	2.31±0.18	2.37±0.10	1.45±0.03

DLS was measured at 0.5 mg/mL, pH 7.5, and 25-90°C, and after cooling the sample back to 25°C after heating. Rh values were calculated from size-number graphs. The errors are the standard deviation of three-times measurements with the same sample.
